# Pilot-Scale Melt Electrospinning of Polybutylene Succinate Fiber Mats for a Biobased and Biodegradable Face Mask

**DOI:** 10.3390/polym15132936

**Published:** 2023-07-03

**Authors:** Maike-Elisa Ostheller, Naveen Kumar Balakrishnan, Konrad Beukenberg, Robert Groten, Gunnar Seide

**Affiliations:** 1 Aachen-Maastricht Institute for Biobased Materials (AMIBM), Maastricht University, Brightlands Chemelot Campus, Urmonderbaan 22, 6167 RD Geleen, The Netherlands; gunnar.seide@maastrichtuniversity.nl; 2 Aachen-Maastricht Institute for Biobased Materials e.V. (AMIBM e.V.), Lutherweg 2, 52068 Aachen, Germany; naveen.balakrishnan@maastrichtuniversity.nl (N.K.B.); konrad.beukenberg@maastrichtuniversity.nl (K.B.); 3 Department of Textile and Clothing Technology, Niederrhein University of Applied Sciences, Campus Moenchengladbach, Webschulstrasse 31, 41065 Moenchengladbach, Germany; robert.groten@hs-niederrhein.de

**Keywords:** melt electrospinning, polybutylene succinate, nonwovens, fiber spinning, environmental sustainability, fiber production, process development, filtration

## Abstract

The COVID-19 pandemic led to a huge demand for disposable facemasks. Billions were manufactured from nonbiodegradable petroleum-derived polymers, and many were discarded in the environment where they contributed to plastic pollution. There is an urgent need for biobased and biodegradable facemasks to avoid environmental harm during future disease outbreaks. Melt electrospinning is a promising alternative technique for the manufacturing of filter layers using sub-microfibers prepared from biobased raw materials such as polybutylene succinate (PBS). However, it is not yet possible to produce sub-micrometer PBS fibers or uniform nonwoven-like samples at the pilot scale, which hinders their investigation as filter layers. Further optimization of pilot-scale PBS melt electrospinning is therefore required. Here, we tested the effect of different parameters such as electric field strength, nozzle-to-collector distance and throughput on the final fiber diameter and sample uniformity during PBS melt electrospinning on a pilot-scale device. We also studied the effect of a climate chamber and an additional infrared heater on the solidification of PBS fibers and their final diameter and uniformity. In addition, a post-processing step, including a hot air stream of 90 °C for 30 s has been studied and successfully lead to a nonwoven-like structure including filaments that weld together without changing their structure. The finest fibers (1.7 µm in diameter) were produced at an applied electric field strength of −40 kV, a nozzle-to-collector distance of 5.5 cm, and a spin pump speed of 2 rpm. Three uniform nonwoven-like samples were tested as filter layers in a medical face mask by measuring their ability to prevent the transfer of bacteria, but the pore size was too large for effective retention. Our results provide insight into the process parameters influencing the suitability of melt-electrospun nonwoven-like samples as biobased and biodegradable filter materials and offer guidance for further process optimization.

## 1. Introduction

Plastics in the environment have become a severe risk to global ecosystems and human health [1]. The COVID-19 pandemic led to a demand for billions of single-use facemasks, which were manufactured from nonbiodegradable and petroleum-derived polymers, resulting in a huge increase in the environmental burden of microplastics [2]. The standard medical facemask has at least three nonwoven fabric layers to protect the wearer. The middle layer is the filter material, preventing the inward and outward transmission of aerosols, whereas the inner and outer layers provide fit and avoid direct skin contact with the filtration layer [3]. The middle layer is usually made from melt-blown fibers with a diameter of 0.5–10 µm whereas the other layers are spun-bonded and the average fiber diameter is 10–50 µm [4]. This results in a filtration efficiency of 95% [1]. Most medical facemasks are manufactured from polypropylene (PP) due to its excellent physical properties and processability [1] but PP takes 20–30 years to fully break down in the environment. Recycling options are limited because facemasks are often contaminated with hazardous microbes. Therefore, most PP facemasks contribute to plastic pollution in landfills, waste dumps, water bodies or littering in public places [1]. These issues have increased the demand for strategies to reduce the environmental impact of medical masks, including the use of biodegradable and biobased polymers [5,6,7].

Melt-blown and spun-bonded fibers tend to be coarse, whereas electrospinning is emerging as a cost-effective, versatile and efficient method for the preparation of fibers in the low micrometer to nanometer range [8,9,10]. These highly flexible fibers feature an enormous surface area, making them suitable for applications in biomedicine [11], filtration and separation [12], textile manufacturing, electronics and energy [13]. Electrospinning involves the creation of an electric field by establishing a potential difference between the end of a needle capillary and a collector [14]. This confers a surface charge upon a polymer solution or melt, resulting in the formation of a so-called Taylor cone. When the electrostatic repulsive force of the surface charge overcomes a specific surface tension, a charged jet stream is ejected from the tip of the Taylor cone. The charge density of the jet interacts with the external field and causes whip-like instabilities, which stretch the fiber and reduce its diameter to the micrometer and even sub-micrometer range. The continuous filaments are deposited as a nonwoven-like material on the collector [15]. Electrospinning allows the fiber-forming process to be tuned by varying process parameters such as the electric field strength, the throughput, and the nozzle-to-collector distance, allowing the production of fibers with diameters of 0.08–0.7 µm, which would be highly beneficial for facemask applications [16].

The two major electrospinning techniques are solution electrospinning and melt electrospinning. Solution electrospinning is more commonly used because the lower viscosity and higher electrical conductivity of polymer solutions leads to significantly narrower fibers [17]. However, only 2–10% of the liquid processed during solution electrospinning is the polymer, the remainder being a toxic solvent that evaporates during processing [14]. The high evaporation rate of the solvent, the strong dependency on the elasticity of the polymer solution, and the low flow rates needed to produce narrow fibers all reduce the productivity of solution electrospinning and thus hinder its industrial application [17]. Melt electrospinning is a more efficient process and there is no need for any solvents, making it a more environmentally sustainable alternative [14]. Research has therefore focused on optimizing the melt-electrospinning process to produce nanoscale fibers, especially from biobased and biodegradable polymers [18,19,20,21,22,23,24].

Biocompatible and biodegradable polymers are optimal in biomedical procedures, especially when used as sutures, bone fixtures, plates, stents, screws, and as tissue repair and tissue engineering matrices [25]. Polybutylene succinate (PBS) has emerged as one of the most promising biobased polymers because it is biodegradable and has excellent melt processability and chemical resistance [26], making it suitable for applications such as compostable bags, nonwoven garments and sheets [27]. A biodegradable face mask filter medium was recently prepared from PBS nanofibers by solution electrospinning [28]. The resulting fiber mat was superior to a filter material prepared from microfibers. Furthermore, it was broken down within 1 month under composting conditions. However, there is a risk of solvents being carried over to the final product, so it would be important to use safe, ecofriendly solvents or switch to melt electrospinning [29].

The melt electrospinning of biopolymers such as PBS has yielded promising results [18,19,30], but we are unaware of any prototype face masks (or filter layers) produced by pilot-scale melt electrospinning. We previously reported the first melt-electrospun PBS fibers in the low micrometer range prepared on a pilot-scale device [18]. The minimum fiber diameter achieved thus far is 10.88 µm [21]. We recently found that changing the polarity of the collector on laboratory-scale and pilot-scale devices influences the properties of PBS fibers, with fiber webs deposited on a negatively charged collector being finer, more densely and uniformly arranged, and covering a larger surface area than those deposited on a positively charged collector. Even so, the fiber webs produced during pilot-scale melt electrospinning did not weld together and resembled a loose web rather than a nonwoven, making them less suitable as filter materials. Given the high cost of machine optimization, cost-effective alternatives are needed to address these constraints. We therefore determined the effect of (1) a climate chamber placed around the spinneret and (2) an additional infrared heating source on the solidification of the PBS melt after leaving the nozzles. We also investigated the influence of additional process parameters (electric field strength, nozzle-to-collector distance and throughput) on the fiber diameter and sample uniformity. The three most homogeneous samples were used to develop medical face mask prototypes that incorporate melt-electrospun nonwovens as a filter layer. We determined the pore size of the filter layer and tested its ability to prevent the passage of bacteria. Our results can be used to facilitate the pilot-scale melt electrospinning of biobased polymers for the sustainable production of narrower fibers and homogenous samples suitable for filtration applications.

## 2. Materials and Methods

### 2.1. Materials

For all experiments, we used biobased PBS fiber-grade resin (FZ78TM) produced by the polymerization of biobased succinic acid and 1,4-butanediol. The manufacturer (MCPP, Düsseldorf, Germany) reported the following specifications: melt flow rate = 22 g/10 min at 190 °C using a weight of 2.16 kg, and a crystalline melting temperature of 115 °C. The polymer was vacuum dried at 60 °C for 12 h before processing.

### 2.2. Melt Electrospinning Equipment

**Figure 1:**
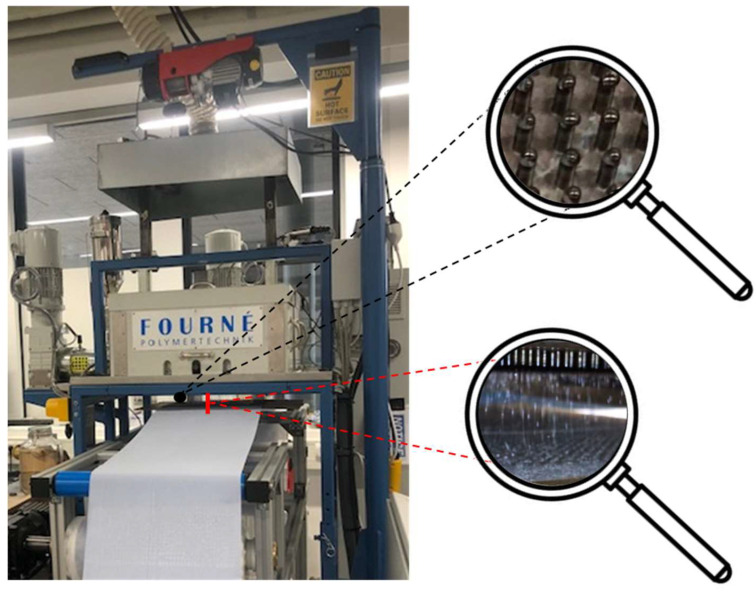
Pilot-scale melt electrospinning prototype including a 600-nozzle plate, showing the placement and design of the individual nozzles (upper magnifying glass); and the filaments ejected by the individual nozzles that are being collected as nonwoven like samples on an aluminum plate (lower magnifying glass).

We used a new prototype pilot-scale melt-electrospinning device developed during a corporation between the Aachen-Maastricht Institute for Biobased Materials (AMIBM) at Maastricht University, Fourné Maschinenbau GmbH (Alfter-Impekoven, Germany) and Poetter-Klima Gesellschaft fuer Nanoheiztechnik mbH (Georgsmarienhuette, Germany). The machine includes a spinneret with 600 nozzles as can be seen in the upper magnifying glass shown in Figure 1, each 0.3 mm in diameter and spaced at 8-mm intervals, which vastly exceeds the capabilities of any state-of-the-art technologies [31]. A speed-adjustable single-screw extruder with three heating zones based on integrated heating elements and a spinning pump ensured a constant supply of polymer melt. Heating elements around the spinneret were used to avoid the blocking of individual nozzles caused by rapid polymer solidification.

Melt electrospinning was carried out at an extruder and spinneret temperature of 235 °C. A 34 × 14 cm aluminum plate served as a collector for the continuous filament deposition of the melt electrospun PBS filaments ejected by the 600 nozzles (shown in the lower magnifying glass in Figure 1), allowing the production of continuous nonwovens. The process parameters are summarized in Table 1. Melt electrospinning was carried out at room temperature (22.7 °C) with a relative humidity of 63%.

We previously reported the low throughput of the pilot-scale device resulting in slow fiber deposition [18]. This gives the PBS sufficient time to solidify after leaving the nozzle but before reaching the collector, so the fibers do not weld together. To overcome this issue, we (1) enclosed the spinneret in a custom-made glass climate chamber or (2) applied an infrared heat source (LGA, Nuernberg, Germany), thus keeping the fibers warm after extrusion to delay crystallization.

### 2.3. Characterization of PBS Granules

The thermal characteristics of PBS was tested using a flash DSC 2+ chip calorimeter (Mettler Toledo, Barcelona, Spain) connected to a TC-100 intracooler (Huber, Offenburg, Germany). The sensor was calibrated, and a sample was heated and cooled twice to ensure proper contact between the polymer and the sensor. Measurements were taken in a nitrogen atmosphere at a flow rate of 80 mL/min to prevent degradation. We used STARe software version 16.00 from Mettler Toledo to analyze the data. To study the melt memory effect, the sample was first heated to 140 °C (25–30 °C above its melting peak) to erase its thermal history. It was then cooled to −60 °C at different rates, ranging from 0.167 K/s to 100 K/s, to obtain a standard crystalline state and was maintained at this temperature for 0.1 s. The rheological properties of PBS were determined using a plate–plate Discovery HR1 hybrid rheometer (TA Instruments, New Castle, DE, USA). The effect of temperature on the complex viscosity of PBS was characterized in a temperature sweep (240–200 °C) with a 25-mm plate at an angular frequency of 10 rad/s.

### 2.4. Characterization of PBS Fibers

Fiber diameters were measured using an Olympus BX53 microscope fitted with an Olympus DP26 camera (Olympus, Leiderdorp, The Netherlands). The fiber diameter was measured 100 times for each sample in different positions, based on the 50× magnified image. The mean flow pore size of the three most homogenous PBS samples was determined by liquid displacement using a PSM 165 pore size meter (Topas, Dresden, Germany) according to ASTM E 1294-89 and ASTM F316-03. Perfluorocarbon (surface tension σ = 16 mN/m) was used as the testing fluid. The air supply was connected with a hose from the test instrument to the sample holder.

### 2.5. Characterization of the PBS Filter Prototype

The bacterial filtration efficiencies (%BFE) of prototype medical face mask materials were compared to a reference according to ASTM Test Method S210-01, along with the recommended positive and negative controls. We tested penetration by an aerosol of *Staphylococcus aureus*, a Gram-positive bacterium with a diameter of 0.5–1.0 µm. The aerosol was collected in the six-stage viable particle cascade impactor. The exposed plates were incubated at 37 °C for 16 h before counting colonies, and the %BFE was calculated as follows:

(1) $$100\frac{C-T}{C}=\%\;BFE$$

where *C* and *T* are the average plate counts for the control and test samples, respectively.

## 3. Results and Discussion

### 3.1. Glass Chamber and Infrared Heating to Delay Polymer Crystallization

**Figure 2:**
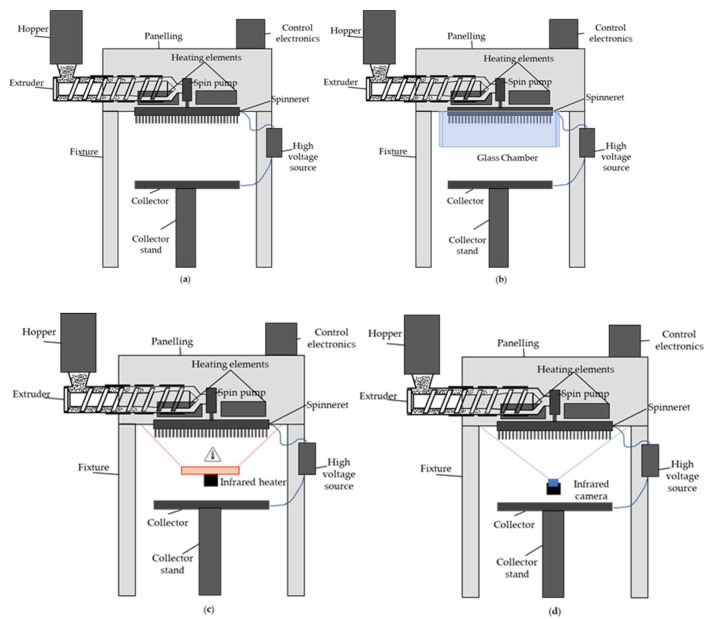
Schematic drawing of the melt-electrospinning setup showing (**a**) the 600-nozzle pilot-scale device; (**b**) the addition of a glass chamber; (**c**) the additional infrared heating source; and (**d**) the position of the infrared camera.

Figure 2a shows a schematic drawing of the pilot scale melt-electrospinning device. To prevent the premature cooling and crystallization of the spun fibers, we used a glass chamber (Figure 2b) and infrared heater (Figure 2c) to maintain a higher temperature.

**Figure 3:**
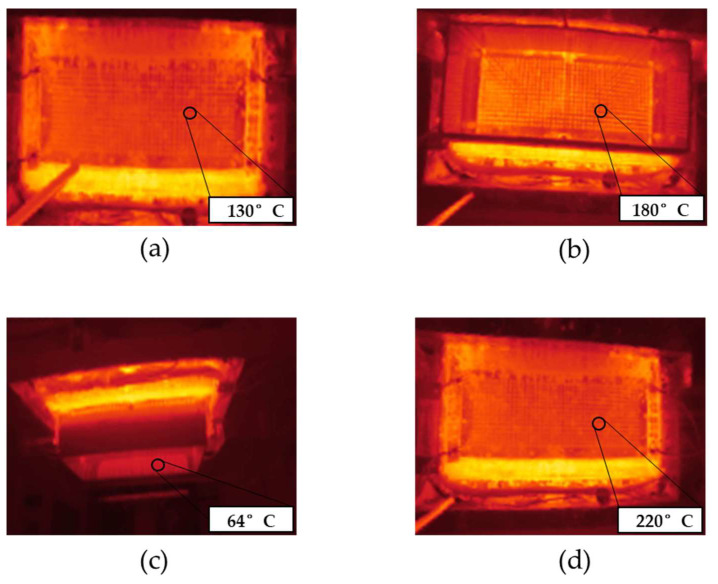
The spinneret of the pilot-scale melt electrospinning device heated to 235 °C. The surface temperature of the nozzles was recorded (**a**) without a glass chamber and (**b**) with a glass chamber. (**c**) The temperature inside the glass chamber. (**d**) The surface temperature of the nozzles in the presence of a supplementary infrared heating device.

Figure 3 shows infrared images of the spinneret at an applied temperature of 235 °C in the presence and absence of the climate chamber and in the presence of a supplementary infrared heater. All images were taken from below the spinnerets, as shown in Figure 2d.

In the absence of the climate chamber or supplementary heating, the surface temperature of the nozzles was 130 °C (Figure 3a). This means the polymer melt cools rapidly from 235 °C inside the extruder and spin pack to ~130 °C inside the nozzles. The 600 nozzles (8 mm in length and 0.3 mm in diameter) are distributed over the spinneret, which has an area of 467 cm^2^ [31]. The spinning plate is heated and the nozzles protrude. The nozzles are therefore cooled by the surrounding air and their surface temperature is lower than that of the spinning plate, which leads to significant heat loss from the polymer melt. When we placed the glass chamber around the spinneret, the surface temperature of the nozzles increased to 180 °C (Figure 3b) and the temperature inside the glass chamber and below the spinneret climbed to 64 °C (Figure 3c). However, this was insufficient to prevent the cooling of the spun fibers, which did not become finer or stickier as anticipated. The temperature of 64 °C inside the chamber and below the spinneret thus appears insufficient to prevent the crystallization of PBS before it reaches the collector. We therefore added an infrared heater, which increased the surface temperature of the nozzles to 220 °C (Figure 3d). There was still no improvement in the processability of the PBS. Indeed, we observed polymer droplets emerging from the nozzles rather than continuous filaments. A temperature of ~220 °C below the spinneret, which is ~100 °C above the melting temperature of PBS, is therefore too high to ensure a melt viscosity sufficient to stretch the melt into filaments. We conclude that neither the presence of a glass chamber nor the provision of supplementary heat was able to achieve a melt viscosity that favors the spinning of narrower fibers and fibers that form a nonwoven-like structure.

### 3.2. Thermal Analysis of PBS

**Figure 4:**
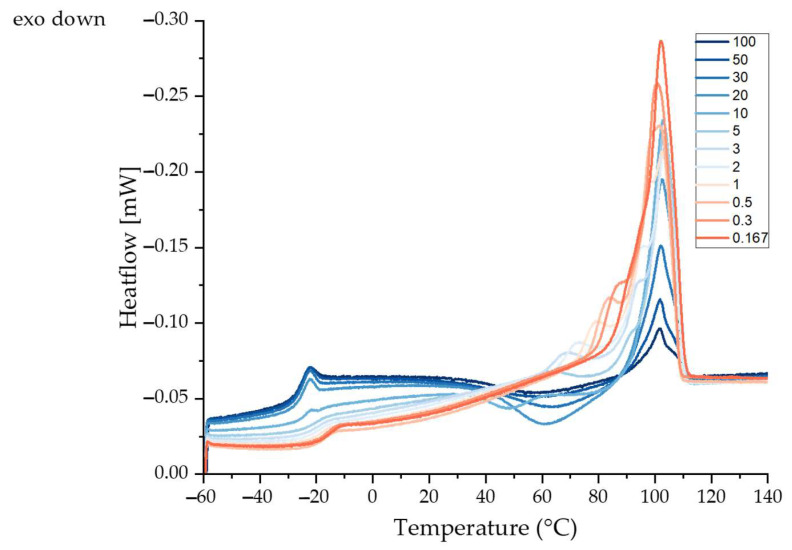
Heating cycle of PBS cooled at different cooling rates from 100 K/s to 0.167 K/s using flash differential scanning calorimetry.

The heating cycle of PBS was assessed by flash differential scanning calorimetry (DSC). Data collected at different cooling rates revealed different exothermic events. The glass transition temperature (T_g_), recrystallization temperature (T_rc_) and additional exothermic peaks (T_m1_, T_m2_ and T_m3_) at different cooling rates can be derived from the curves shown in Figure 4. The T_g_ increased slightly with slower cooling, probably reflecting the formation of more crystalline regions in the polymer. Crystalline regions form physical cross-links due to hydrogen bonding, restricting the mobility of the polymer chains in the amorphous regions and thus increasing the T_g_ [32,33]. The T_g_, T_rc_ and T_m_ values are summarized in Table 2.

Exothermic peaks (T_rc_) were apparent at all cooling rates, probably reflecting the melt recrystallization of crystallites with poor thermal stability [34]. The endothermic peak at ~102 °C (T_m1_) was independent of the cooling rate, but the enthalpy increased with slower cooling. A possible explanation for the higher enthalpy at low cooling rates is that faster cooling reduces the opportunity for crystals to form, resulting in a lower degree of crystallinity [35,36]. Slower cooling rates (5–0.167 K/s) produce an additional shoulder peak (T_m2_) at ~95 °C that probably reflects the melting of different types of crystals or the formation of defective crystals [34]. PBS hat two crystalline forms (alpha and beta) and the beta form only exists under strain. Slow cooling may induce stress, resulting in the formation of beta crystals that generate the additional melting peak. A third peak (T_m3_) appeared at cooling rates of 5–0.5 K/s. This may be an annealing peak representing the transition of the rigid amorphous fraction (RAF) from a solid-like to a liquid-like state [34].

We conclude that PBS crystallizes regardless of the cooling rate, but higher rates lead to a lower degree of crystallinity and lower rates induce a stress state that favors the formation of beta crystals. We previously showed that PBS starts to crystallize at ~80 °C [19]. Therefore, in order to stretch the fibers during the melt electrospinning process, the temperature below the spinneret must remain above 80 °C. The DSC data show that the addition of the glass chamber, and the resulting temperature of ~64 °C, is insufficient to prevent the crystallization of PBS filaments before they reach the collector.

### 3.3. Effect of Temperature on Viscosity

**Figure 5:**
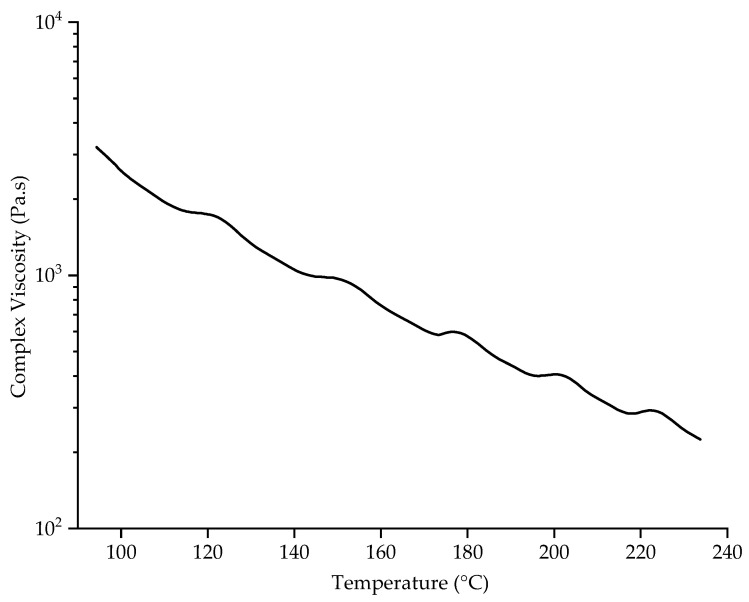
Rheogram showing the complex viscosity of PBS as a function of temperature at an angular frequency of 10 rad/s.

The rheological properties of PBS were determined by measuring its complex viscosity as a function of temperature at an angular frequency of 10 rad/s (Figure 5).

PBS was previously reported to have a complex viscosity of 131.56 Pa.s at a spinning temperature of 235 °C [30]. The complex viscosity increases at lower temperatures, most likely due to entanglements and chain interactions that break at high temperatures [30]. We found that the complex viscosity of PBS was 1337 Pa·s at 130 °C, the temperature of the nozzle surface at a spinneret temperature of 235 °C. This is 10-fold higher than the preferred viscosity of 131.56 at 235 °C, and with the absence of a heating chamber between the nozzle and collector the fibers cool very quickly and the polymer melt does not have enough time to be drawn by the electric field to produce narrow fibers [18]. We previously found that the electrical resistance of the PBS melt was ~8 GΩ at 145 °C and that higher temperatures generally reduced the electrical resistance. Increasing the polymer melt temperature to 235 °C therefore reduced the electrical resistance by 10-fold, which facilitates interactions with the electric field and reduces the fiber diameter [30]. Additional heating is therefore required around the spinneret to reduce the complex viscosity and increase the electrical conductivity of the PBS melt, facilitating jet thinning before fibers reach the collector. By adding a glass chamber around the spinneret, the temperature of the nozzles increased to 180 °C, and the temperature underneath the spinneret reached ~64 °C. The ~50 °C increase at the nozzle reduced the complex viscosity of PBS to 562 Pa·s, which is still relatively high. Furthermore, the temperature underneath the spinneret was still too low to improve the spinnability of the PBS melt and allow the fibers to weld together on the collector. Supplementary infrared heating increased the nozzle temperature to ~220 °C, reducing the complex viscosity to 291 Pa·s and causing the melt to drip from the nozzles. This complex viscosity therefore appears unable to draw the filaments into narrow fibers. The ambient temperature of 220 °C around the nozzle is ~100 °C above the melting temperature of PBS, so the polymer is likely to be completely molten and drawing into filaments is no longer possible, resulting in the polymer dripping. We conclude that neither the glass chamber nor the infrared heater achieved a sufficient temperature beneath the spinneret to maintain an appropriate complex viscosity once the molten polymer had left the nozzles. Future research should test a heating device that maintains a nozzle temperature of 235 °C while keeping the surrounding air above the crystallization temperature but far below the melting temperature. This might achieve a complex viscosity that promotes whipping behavior and jet elongation, prolonging interactions with the electric field, delaying polymer solidification, and thus producing sticky fibers that form a nonwoven-like structure.

### 3.4. Fiber Diameter and Distribution

**Figure 6:**
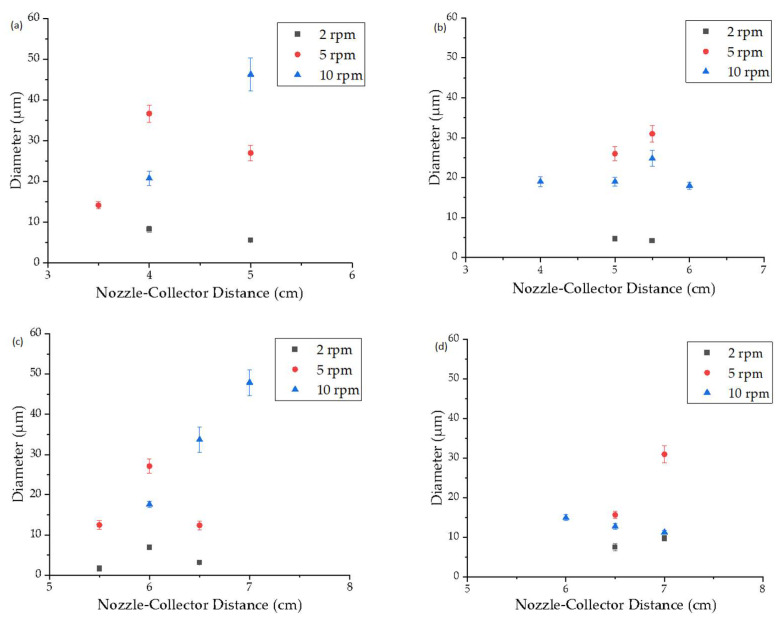
Diameter of PBS fibers produced using a pilot-scale melt electrospinning device with a spinneret temperature of 235 °C, a spin pump speed of 2, 5 or 10 rpm, a nozzle-to-collector distance of 3.5, 4, 5, 5.5, 6, 6.5 or 7 cm, 600 nozzles (0.3 mm diameter), and an electric field strength of (**a**) −30 kV, (**b**) −35 kV, (**c**) −40 kV and (**d**) −45 kV. Data are means ± standard deviation (*n* = 100).

The average fiber diameter was evaluated at an extruder and spinneret temperature of 235 °C while varying the spin pump speed, nozzle-to-collector distance, and electric field strength (Figure 6). Regardless of the electric field strength, a spin pump speed of 2 rpm produced the narrowest fibers at all nozzle-to-collector distances and the fiber diameter increased with increasing spin pump speed, consistent with our previous report [18]. When the electric field strength was low (−30 and −35 kV) small nozzle-to-collector distances of 3–5 cm showed enhanced whipping behavior and a good interaction between the polymer jet and the electric field. With greater distance between the nozzle and collector, the fiber diameter increased due to the lower electric field strength, which limited interactions between the polymer jet and electric field [30]. Similar observations have been made by researchers in the past where increasing the nozzle-to-collector distance during the melt-electrospinning of PLA led to an increase in fiber diameter [20]. At higher electric field strengths (−40 and −45 kV), nozzle-to-collector distances of 3–5 cm resulted in short circuits, observed as flashes between the nozzle and collector, indicating that the electric field strength was high enough for electrons to jump directly from one side to the other.

**Figure 7:**
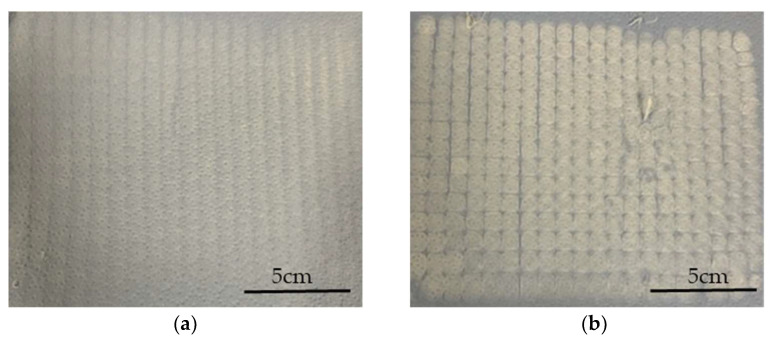
Fiber web samples produced using the pilot-scale melt electrospinning device at an applied field strength of −35 kV, a pump speed of 5 rpm, and a nozzle-to-collector distance of 5 cm. (**a**) Sample produced by the slow clockwise rotation of the thin paperboard placed above the collector. (**b**) Sample produced without the slow clockwise rotation of the thin paperboard placed above the collector.

Increasing the electric field strength had a significant effect on the fiber diameter at all spin pump speeds. The finest fibers (diameter 1.7 µm) were achieved at an applied electric field strength of −40 kV, a nozzle-to-collector distance of 5.5 cm and a spin pump speed of 2 rpm, which is the finest diameter achieved for PBS on a pilot-scale melt-electrospinning machine so far, coming close to the smallest average fiber diameter of 810 nm, achieved during pilot-scale melt-electrospinning with PLA [31]. It was previously established that changing the polarity of the collector from +30 to −30 kV achieves a more uniform distribution of fibers [18]. Contrary to our expectations, increasing the electric field strength did not further affect the uniformity of the sample, regardless of the spin pump speed and nozzle-to-collector distance. Therefore, we investigated the rotation of the thin paperboard placed above the collector. Slow clockwise movement had a greater influence than adjusting the process parameters, and led to a significant improvement in the homogeneity of fiber deposition (Figure 7). These results suggest that changing process parameters such as the nozzle-to-collector distance, the electric field strength, and the spin pump speed does not improve the uniformity of the sample further, but different collector designs, mechanisms and rotation speeds should be investigated in the future to determine their effects.

### 3.5. Prototyping

**Figure 8:**
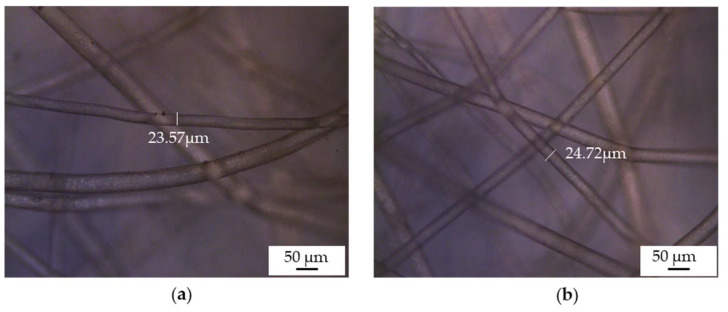
Optical microscopy images of PBS melt-electrospun fibers. (**a**) Sample before heat processing. (**b**) Sample after heat processing.

Before prototyping, the melt-electrospun samples were post-processed to make the filaments weld together. We applied a hot air dryer for 30 s at a temperature of 90 °C, which is ~15 °C below the melting temperature of PBS. Figure 8 shows a PBS sample, spun at an applied voltage of −35 kV, a nozzle-to-collector distance of 5 cm and a spinpump speed of 5 rpm that serves as a filter for prototype mask 3, before and after processing, revealing adhesion points in the post-processed sample. The selected temperature warmed the filaments enough to promote adhesion without changing their structure, so there was no significant change in fiber diameter. The chosen post-processing process was found to be adequate for PBS and solved the problem of a lose fiber web formation.

**Figure 9:**
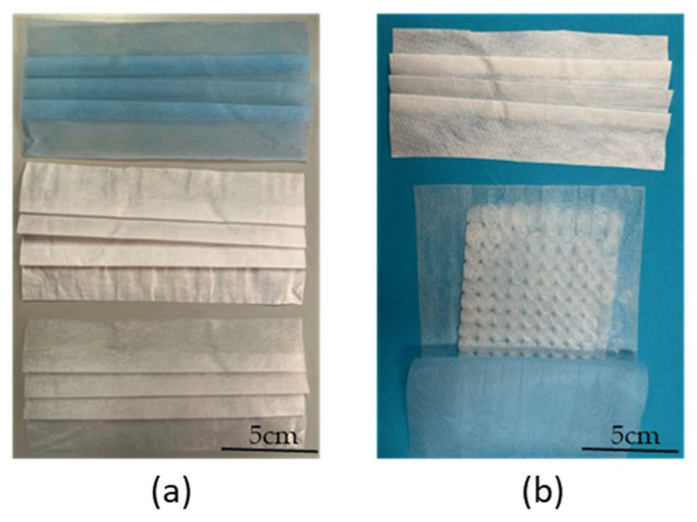
Comparison of a commercial medical face mask and a prototype mask incorporating a melt-electrospun PBS filter layer. (**a**) The disassembled reference medical face mask. (**b**) The layers of the prototype mask before assembly.

Figure 9 shows a commercial medical face mask (used as a reference) and a prototype face mask containing melt-electrospun PBS as a filter layer. The prototype was created by disassembling a commercial medical face mask, removing the middle layer that acts as a filter, and replacing it with our melt-electrospun PBS sample.

**Figure 10:**
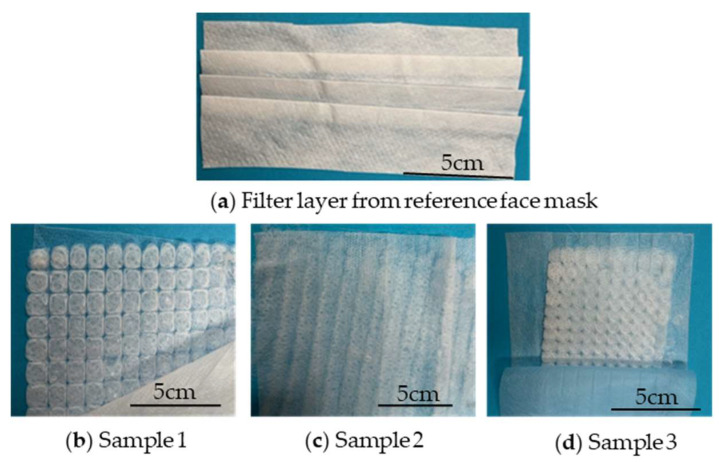
Comparison of the filter layers of (**a**) the commercial medical face mask and (**b**–**d**) three prototypes based on melt-electrospun PBS samples.

Figure 10 shows the three melt-electrospun PBS samples used as filter material in the prototype masks compared to the filter layer of the commercial medical face mask.

### 3.6. Bacterial Filtration Efficiency of the Prototypes

We tested the %BFE of four masks: the commercial medical mask as a reference, and three prototypes with nonwoven-like PBS samples produced using our pilot-scale melt electrospinning device. Table 3 summarizes the manufacturing details of the prototypes and the product details of the reference material.

The %BFE for each mask is presented in Table 4.

**Figure 11:**
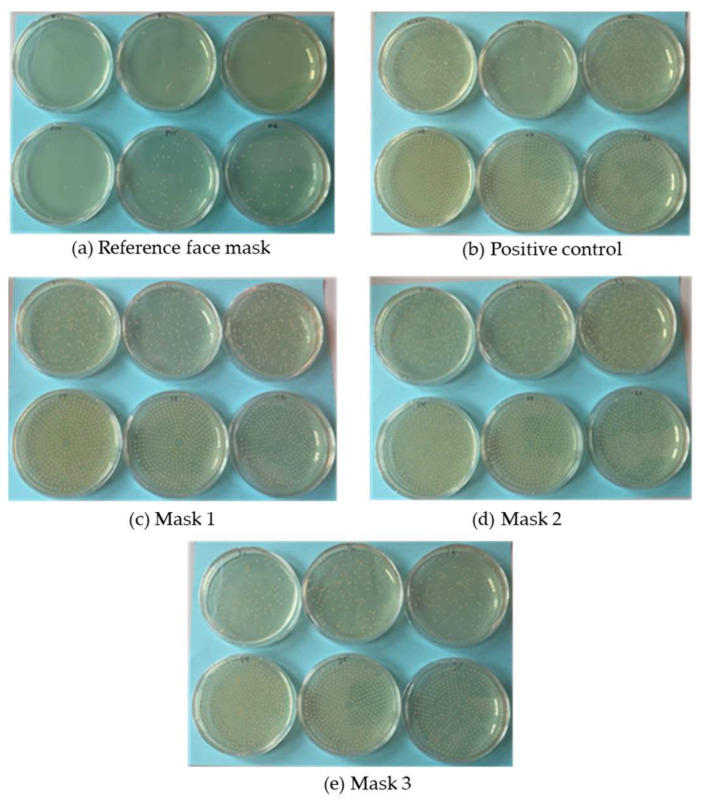
Plate images to provide a visual representation of the differences in bacterial filtration efficiency between reference and prototype masks. (**a**) The reference face mask. (**b**) Positive control according to ASTM Test Method S210-01. (**c**–**e**) The three prototype masks.

The %BFE for the reference mask was significantly higher than that of all three prototypes based on the ~82-fold increase in the number of colony forming units (CFUs) on plates tested with the prototype masks (Table 4). The plates are shown in Figure 11. We expected the prototypes to achieve a lower %BFE than the commercial face mask because the PBS nonwoven-like samples were not as homogenous as the commercial material (Figure 10). However, the %BFE values for the prototypes were still lower than we anticipated. Previous studies have demonstrated a relationship between pore size and %BFE, with large pore sizes corresponding to poor filtration efficiency [37]. The low %BFE values for all three prototypes therefore suggest that the PBS nonwovens have a large pore size.

### 3.7. Pore Size Measurements

The pore size distribution of the commercial face mask (reference material) is shown in Table 5.

For the reference mask, the cumulative share of pores in the 13.2–15.5 µm size range was 99.2% with the remaining 0.08% in the 15.5–17.8 µm size range. In contrast, all PBS samples exceeded the upper limit of the measurement range (500 µm) suggesting the pores were at least ~33-fold larger than those in the commercial material.

## 4. Conclusions and Outlook

We produced the first prototype melt-electrospun PBS samples using our pilot-scale melt electrospinning device. We applied two machine modifications to overcome the rapid solidification of PBS, which allows less time for whipping and interaction with the electric field thus leading to coarse, nonadhesive PBS fibers. The first approach was the integration of a glass chamber, which did increase the temperature beneath the spinneret, but the filaments still did not weld together. The second approach was the integration of an infrared heater, which increased the temperature further but caused the formation of polymer droplets. Flash DSC and rheological [14] analysis confirmed that the temperature remained too low following the integration of the glass chamber and too high with supplementary infrared heating for effective melt electrospinning. Furthermore, the temperature of the nozzle surface remained below 235 °C. One possible solution is therefore the integration of a heating device that maintains a nozzle temperature of 235 °C while keeping the temperature beneath the spinneret above the crystallization temperature but well below the melting point of PBS. However, including a post-processing step of applying a hot air dryer for 30 s at a temperature of 90 °C to the polymer samples, has led to the successful welding of the single filaments and obtained a nonwoven-like structure without changing their structure. Although increasing the electric field strength did not significantly improve the homogeneity of our samples, it reduced the final fiber diameter to ~1.7 µm, which is the finest PBS fiber produced by melt electrospinning thus far. Even though, it is still challenging to reach the nanometer range during the pilot-scale melt-electrospinning of PBS yet, as achieved during former research with PLA (810 nm) [31], the experiments have provided a valuable insight into the direction of biodegradable and biobased filter materials for face mask applications. The prototype face masks achieved much lower %BFE ratings than a commercial medical face mask, reflecting the much larger pore size in the PBS nonwoven-like samples. The slow clockwise rotation of the thin paperboard placed above the collector had the greatest impact on uniformity, and different collector designs and methods should therefore be investigated to improve uniformity and the %BEF. The inclusion of additives such as curcumin or silver confers antibacterial properties and could be investigated for bacterial filtration applications. Although our prototypes were not suitable as filter layers in face masks, further adjustments such as different collector positions, additional heating elements and the inclusion of additives may reduce the fiber diameter and increase the uniformity and %BFE of melt-electrospun PBS samples, which will finally allow the use of melt-electrospun PBS fiber mats for filtration applications.

## Figures and Tables

**Table 1:** Process parameters for the pilot-scale melt electrospinning experiments (without climate chamber or supplemental heating).

Trial	Spin Pump Speed (rpm)	Nozzle–Collector Distance (cm)	Electric Field (kV)
5–10	2	3, 3.5, 4, 5, 5.5, 6	−30
11–12	2	5, 5.5	−35
13–15	2	5.5, 6, 5.5	−40
16–18	2	6.5, 7, 7.5	−45
19–21	5	3.5, 4, 5	−30
22–24	5	5, 5.5, 6	−35
25–27	5	5.5, 6, 6.5	−40
28–29	5	6.5, 7	−45
30–31	10	4, 5	−30
32–37	10	3, 3.5, 4, 5, 5.5, 6	−35
38–40	10	6, 6.5, 7	−40
41–44	10	5.5, 6, 6.5, 7	−45

**Table 2:** The glass transition temperature (T_g_), recrystallization temperature (T_rc_) and melting peaks (T_m1_, T_m2_ and T_m3_) of PBS determined at different cooling rates.

Cooling Rate (K/s)	T_g_	T_rc_ (°C)	T_m1_ (°C)	T_m2_ (°C)	T_m3_ (°C)
100	−20	60	101	-	-
50	−20	60	101	-	-
30	−20	60	102	-	-
20	−20	60	102	-	-
10	−20	48	102	-	-
5	−15	77	102	92	62.8
3	−15	78	102	94.3	68.6
2	−15	80	102	95.6	72.6
1	−15	82	102	97.9	79.4
0.5	−15	85	102	99.2	84.1
0.3	−12	88	102	87.17	-
0.167	−12	-	102	94.5	-

**Table 3:** Manufacturing and product details of the reference (mask 1) and prototypes (masks 2–4).

Mask	Manufacturing and Product Details
1	Nonsterile disposable medical face mask (Europapa) Three layers: outer hydrophobic nonwoven layer, inter melt-blown filter layer and inner soft absorbent layer BFE > 98%
2	7 cm nozzle-to-collector distance 10 rpm spin pump speed −45 kV applied voltage
3	5 cm nozzle-to-collector distance 5 rpm spin pump speed −35 kV applied voltage collector rotated
4	5.5 cm nozzle-to-collector distance 10 rpm spin pump speed −35 kV applied voltage

**Table 4:** Bacterial filtration efficiency (%BFE) of the reference face mask and the three prototypes.

Mask	%BFE	Colony Forming Units (CFUs)
1	98.80	48
2	4.5	4372
3	13.3	3969
4	13.9	3942

**Table 5:** Pore size distribution of the reference face mask.

Lowest Pore Size (µm)	Medium Pore Size (µm)	Upper Pore Size (µm)	Share Cumulative (%)
13.2	14.4	15.5	99.2
15.5	16.7	17.8	0.8
17.8	19.0	20.1	0.0
20.1	21.3	22.4	0.0
22.4	23.5	24.7	0.0
24.7	25.8	27.0	0.0
27.0	28.1	29.3	0.0
29.3	30.4	31.6	0.0
